# Formulation and Characterization of Carvedilol Leciplex for Glaucoma Treatment: In-Vitro, Ex-Vivo and In-Vivo Study

**DOI:** 10.3390/pharmaceutics10040197

**Published:** 2018-10-21

**Authors:** Doaa H. Hassan, Rehab Abdelmonem, Menna M. Abdellatif

**Affiliations:** Department of Pharmaceutics and Industrial Pharmacy, College of Pharmacy, Misr University for Science and Technology (MUST), Giza 12566, Egypt; doaa.hassan@must.edu.eg (D.H.H.); drrahoba@yahoo.com (R.A.)

**Keywords:** carvedilol, Leciplex, Intraocular pressure, glaucoma

## Abstract

This study evaluated the efficacy of cationic nanoparticle (leciplex) to deliver carvedilol to ocular surface for glaucoma treatment as recent studies pointed out the effect of topical carvedilol on intraocular pressure, therefore carvedilol loaded leciplex formulae were prepared using soy phosphatidyl choline (SPC) and cationic surfactant (CTAB/DDAB) and characterized for morphology, entrapment efficiency, particle size, zeta potential and ex-vivo corneal permeation. Then the selected formula was evaluated via in-vivo studies in comparison with carvedilol solution. Leciplex nanoparticles appeared spherical in shape with entrapment efficiency exceeded 95% in all formulae. Leciplex formula composed of SPC and DDAB in (1:1) molar ratio showed the smallest particle size (16.04 ± 1.2 nm), highest zeta potential value (53.9 ± 0.91 mv) and highest apparent corneal permeability coefficient (0.1157 cm/h). Carvedilol leciplex reduced intraocular pressure (IOP) to normal range in ocular hypertensive rabbits after 30 min and duration of action lasted for 24 h, while carvedilol solution reduced IOP to normal value after 60 min and duration of action lasted for 6 h. Furthermore, histological examination of eyeballs of rabbits treated with carvedilol leciplex showed improvement of retinal atrophy of glaucomatous eyes. This study concluded that leciplex improve transcorneal permeation and bioavailability of carvedilol.

## 1. Introduction

Carvedilol is a beta-adrenergic blocking agent, indicated for the treatment of hypertension, angina pectoris, and heart failure [1]. Recent study suggests carvedilol as possible therapy for the increased intraocular pressure [2]. The intraocular pressure is controlled by the flow of aqueous humor in the eye where the excess fluid is drains through the trabecular meshwork. If the outflow is blocked, aqueous humor accumulates inside the eye leading to increased intraocular pressure (IOP). The increased IOP can damage the optic nerve, resulting in an optic neuropathy and irreversibly impaired vision [3]. 

Topical eye drop is usually selected for the treatment of eye diseases as it is the most desirable dosage form. However, traditional eye drop usually suffers from low bioavailability due to the limited eye capacity, blinking reflex, lachrymal fluid erosion and nasolacrimal drainage. Also corneal and conjunctival epithelia of human eye, along with the tear film, construct a compact barrier preventing the drug absorption into the intraocular area which shorten the retention time in the eyes and produce less absorption in the intraocular area [4], therefore, frequent instillation of eye drops is often required to achieve expected therapeutic efficacy but patients’ long term adherence to installation schedules is a major issue [5].

The use of colloidal drug delivery systems such as liposomes, niosomes, microemulsion, nanoemulsion and nanoparticles in ophthalmic drug delivery have been widely studied. Advantages of colloidal carriers include sustained and controlled release of the drug at the targeted site, reduced frequency of administration, ability to overcome blood–ocular barriers, and efflux-related issues associated with the parent drug and ease of administration as it can be applied in liquid form just like eye-drops solutions. Thus, they avoid the discomfort that results from the application of viscous or sticky preparations [6]. Despite a large variety of colloidal carriers in the ophthalmic drug delivery field, nanoparticles attract most of the attention as it appears to have greater efficacy over other existing formulations, due to the increased specific area of nanometric sized particles [7].

Several attempts have been made to prolong the preocular retention, improve the absorption of the drug and increase patient compliance by reducing the number of required administrations [8]. One of these are the use of electrostatic interaction between the negatively charged sialic acid moieties present in the mucus membrane on corneal surface and the positively charged drug carrier such as cationic liposomes, cationic nanoemulsion, chitosan coated liposomes and cubosomes, resulting in enhanced corneal retention, reducing drug elimination by lachrymal flow and enhanced transcorneal flux.

Leciplex is self-assembled phospholipid based cationic nanocarriers for the improved delivery of hydrophobic drugs. The advantage of leciplex over traditional vesicular systems is in simplicity of preparation as it is a one-step fabrication process that yields nanosized vesicular systems by simple mixing [9]. The major components of a leciplex system are soy phosphatidylcholine (SPC), a cationic agent and a bio-compatible solvent like Transcutol. 

Leciplex improve oral delivery of hydrophobic drugs as quercetin where cationic nanocarriers are known to increase the permeability and uptake of the drug associated with them [10]. Also, cationic nanocarriers have greater bioadhesive properties due to electrostatic interaction with gastrointestinal mucosa which would result in sustained delivery and eventually a greater therapeutic effect [11].

The current study is focused on screening efficacy of leciplex as ocular drug delivery system, therefore carvedilol leciplex were prepared and evaluated. Ex-vivo and in-vivo permeation studies were conducted and reduction of IOP was assessed in comparison with carvedilol solution.

## 2. Materials and Methods

### 2.1. Materials

Carvedilol was kindly gifted by Global Nabi pharmaceuticals company, Egypt, highly purified diethylene glycol monoethyl ether (Transcutol^@^ HP) was obtained from Gattefosse India Ltd., Mumbai, India, potassium dihydrogen phosphate, disodium hydrogen phosphate and sodium chloride were purchased from El-Nasr Pharmaceutical company, Egypt, soy phosphatidylcholine, cetyltrimethylammonium bromide (CTAB) and dimethyldidodecylammonium bromide (DDAB) were purchased from Sigma-Aldrich, St. Louis, MO, USA. All other reagents and solvents were of HPLC analytical grade obtained from Fisher Scientific company, Waltham, MA, USA.

Albino new zealand rabbits without any ocular damage or diseases were obtained from Misr university for science and technology animal center (Giza, Egypt). All animal studies were approved by the ethical committee of Misr university for science and technology.

### 2.2. Formulation of Carvedilol Leciplex

Soy phosphatidylcholine and the cationic surfactants (CTAB/DDAB) were used in a 1:1 or 5:1 molar ratio as shown in Table 1. Soy phosphatidylcholine and the cationic surfactants were dissolved in Transcutol HP (0.5 mL) by heating at 70 °C in a water bath then 50 mg of carvedilol was added after which 9.5 mL distilled water kept at 70 °C was added with cyclomixing until uniform yellow dispersion was formed [9]. 

### 2.3. Evaluation of Carvedilol Leciplex

#### 2.3.1. Determination of Entrapment Efficiency (EE)

One milliliter of carvedilol leciplex was centrifuged at 20,000 rpm for 1 h at 4 °C using a cooling centrifuge (Sigma 3K 30, Osterode am Harz, Germany). Following centrifugation, the sediment was lysed using 5 mL of methanol and sonicated for 10 min then analyzed at 242 nm using UV-Vis spectrophotometer (Shimadzu UV 1650 Spectrophotometer, Kyoto, Japan). Carvedilol entrapment was determined using the following equation:

 EE%= (ED/TD) ×100 

where EE% is the percent encapsulation efficiency, ED is the concentration of entrapped drug and TD is the total drug concentration.

#### 2.3.2. Particle Size, Distribution and Zeta Potential

Particle size (PS), polydispersity index (PDI) and Zeta potential (ZP) for leciplex formulae were measured by the dynamic light scattering (DLS) technique at 25 °C using Zetasizer (Malvern Instruments, Malvern, UK). The leciplex dispersion was properly 100-fold diluted with purified water. Means and standard deviations were calculated for triplicates measurements.

#### 2.3.3. Morphology

The morphology of leciplex was analyzed using a transmission electron microscope (TEM) (JEM-1230, Joel, Tokyo, Japan). Samples were placed on the surface of carbon coated grid and negatively stained with a 1% aqueous solution of phosphotungstic acid and dried at room temperature prior to visualization [12].

### 2.4. Ex-Vivo Corneal Permeation Study

Ex-vivo corneal permeation studies were carried out using modified franz’s diffusion cell with a diffusion area of 0.785 cm^2^. Fresh cow cornea was fixed between the donor and receptor compartments. Accurately measured 200 µL of leciplex dispersion, equivalent to 1000 µg of carvedilol, were placed in the donor cells. The receptor compartment filled with 10 mL of phosphate buffer saline solution (pH 7.4) containing 20% propylene glycol to ensure sink condition and maintained at 35 ± 1 °C under magnetic stirring at 100 rpm. At appropriate time 0.5 mL of permeation media was withdrawn and an equal volume of fresh media was added into the receiver cell. The samples were filtered through a 0.45 µm membrane and analyzed by using a validated HPLC method. Carvedilol solution was used as a control and the results were reported as the mean of three runs. The amount of drug permeating through the corneal epithelium was plotted versus time, and the apparent corneal permeability coefficient (cm/h) was determined according to the equation: Papp = J_ss_/C_0_ where J_ss_ (steady stat flux) is the slope of the linear portion (µg/hr·cm^2^) and C_0_ is the initial drug concentration (µg/cm^2^).

### 2.5. In-Vivo Evaluation of Carvedilol Leciplex

#### 2.5.1. Pharmacokinetic Study

The rabbits were randomly divided into the following two groups: a group treated with the carvedilol solution and a group treated with the selected carvedilol leciplex dispersion. The rabbits were kept under anesthesia throughout the experiment using sodium pentobarbital (30 mg/kg) injected into the marginal ear vein. After administration of 100 μL carvedilol solution or leciplex formulation (both 5.0 mg/mL), 100 μL aqueous humor was extracted with 1 mL insulin needle after 15, 30, 60, 120, 240, and 360 min and placed in a centrifuge tube. Protein was precipitated by vortex mixing with 0.5 mL methanol. Precipitated protein was removed by centrifugation at 10,000 rpm for 10 min, and the concentration of drug in the supernatant was determined by HPLC. The pharmacokinetic parameters were calculated by noncompartmental method using the WinNonlin pharmacokinetic software (Certara Inc., Princeton, NJ, USA).

#### 2.5.2. Pharmacodynamic Study

The rabbits were topically anesthetized with paclitaxel eye drops. Dexamethasone (0.025%) was dissolved into saline and injected into limbus for each eye. The IOP was measured by an ophthalmotonometer (SW-500, Shanghai, China). If the IOP was higher than the upper limit of the normal IOP of 24.4 mmHg and continued for 1 week, the model was considered successful. Rabbits with high IOP were randomly assigned into two groups (six rabbits per group) where the first group (GP1), the left eye only of each rabbit was treated with single dose 100 μL carvedilol solution and the right eye was treated with saline solution and considered as sham control. The second group (GP2) the left eye only of each rabbit was treated single dose 100 μL carvedilol selected leciplex formula and the right eye was treated with saline solution and considered as sham control. The IOP was measured for 2 days and effects of lowering IOP were compared.

#### 2.5.3. Histological Examination

Following the pharmacokinetic and pharmacodynamic studies, the rabbits were sacrificed by injecting phenobarbital sodium to marginal vein, then eyeballs were removed and placed either in 10% formalin. Tissue specimens were trimmed off, washed and dehydrated in ascending grades of alcohol. The dehydrated specimens were then cleared in xylene, embedded in paraffin blocks and sectioned at 4–6 µm thick. The obtained tissue sections were deparaffinized using xylol and stained using hematoxylin and eosin (H&E) for histopathological examination through the electric light microscope [13].

### 2.6. Statistical Analysis of Data

To investigate the significance difference between the results of studied formulae, the one-way analysis of variance (ANOVA) test was used. The level of significance was set at 0.05, and (*p* < 0.05) was considered to be statistically significant.

## 3. Results and Discussion

### 3.1. In-Vitro Evaluation of Carvedilol Leciplex

#### 3.1.1. Entrapment Efficiency

There was no significant difference between encapsulation efficiency of different leciplex formulae (*p* > 0.05), the encapsulation of hydrophobic drug carvedilol (log P 3.22) exceeded 90% in all formulae as shown in Table 2, suggesting association of the drug with lipid phase.

#### 3.1.2. Particle Size, Distribution and Zeta Potential

The particle size is a crucial factor affecting ophthalmic formulations. Generally, the particle size of nanocarriers that are able to penetrate through the cornea should be smaller than 200 nm [14]. Also, smaller particles are better tolerated by patients than larger particles [15]. Leciplex formulae formulated with 1:1 molar ratio SPC to cationic surfactant revealed particle size less than 100 nm and lower PDI value (<0.2) as shown in Table 2. Leciplex formulae with 5:1 molar ratio revealed particle size higher than 500 nm and higher PDI value. Leciplex dispersion prepared without cationic surfactant showed particle size above 1000 nm. These results indicated the critical effect of cationic surfactant concentration on leciplex particle size. On the contrary of previous studies that demonstrated that double tail cationic surfactant (DDAB) containing leciplex showed mean particle size greater than single tail cationic surfactant (CTAB) leciplex [9,10]. In our study DDAB leciplex formulations revealed smaller particle size than CTAB leciplex formulations (*p* < 0.05), these results might be attributed to stabilizing property of DDAB which was demonstrated by the high surface charge obtained by DDAB leciplex compared to CTAB leciplex. The high surface charge was correlated with decreased aggregation and fusion of nanoparticle and led to subsequent decrease in particle size. These results are in agreement with Varghese et al. who found that particle size of lecithmer formulations was dependent upon stabilizing properties of cationic agent (DDAB/DOTAP). As increasing surface charge led to increase stabilizing properties with subsequent reduction in particle size [16]. 

All DDAB/CTAB leciplex formulae revealed positive surface charge (+31.6 to +53.9 mv) indicating good colloidal stability, the preferential of positive charge for these formulae is to promote the electrostatic interaction between cationic nanovesicles and the negatively charged sialic acid residues of corneal mucins [17]. DDAB formula (F2) showed highest zeta potential value (53.9 ± 0.91) indicating stabilizing effect of cationic surfactant DDAB as discussed before. Formulae (F5 and F6) which was formulated without cationic surfactant revealed low zeta potential value +2.82 ± 0.54 and 7.83 ± 0.63, respectively, the positive surface charge of these formulae might be explained by the fact that carvedilol has (pKa = 7.9). Therefore, the drug would carry positive charge at physiological pH [18].

#### 3.1.3. Morphology

**Figure 1:**
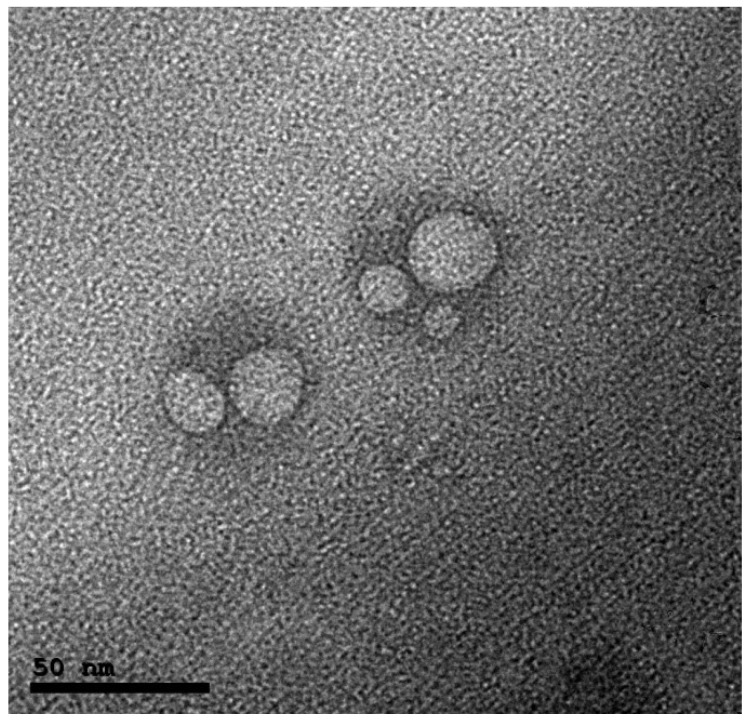
Transmission electron microscope (TEM) images of the carvedilol leciplex (F2).

The TEM micrographs of carvedilol leciplex formula (F2) showed that the leciplex nanovesicles have almost spherical shape as shown in Figure 1. It also confirmed the particle size results obtained by particle size analysis. 

### 3.2. Ex-Vivo Corneal Permeation

Cationic positively charged nanovesicles are not likely penetrate the cornea but rather than bind to the negatively charged mucus [19]. Therefore, the transcorneal drug delivery is probably related to a passive diffusion linked to the enhanced retention time. Also, vesicles size affects drug permeation as the smaller the vesicles size is, the greater interfacial area available for drug exchange and consequently improve clinical efficacy of the drug [20]. These results explained the high apparent corneal permeability coefficient (0.1157 cm/h) obtained by leciplex formula (F2) which showed the smallest mean particle size (16.04 ± 1.2 nm) and highest value of zeta potential (53.9 ± 0.91 mv). Where (F2) provide cumulative drug permeated after 8 h (Q_8_) 480.36 ± 0.58 µg/cm^2^ which was 2.38 times higher than that of drug solution as shown as in Table 3. Therefore, carvedilol leciplex formula (F2) was chosen for in-vivo evaluation. 

**Figure 2:**
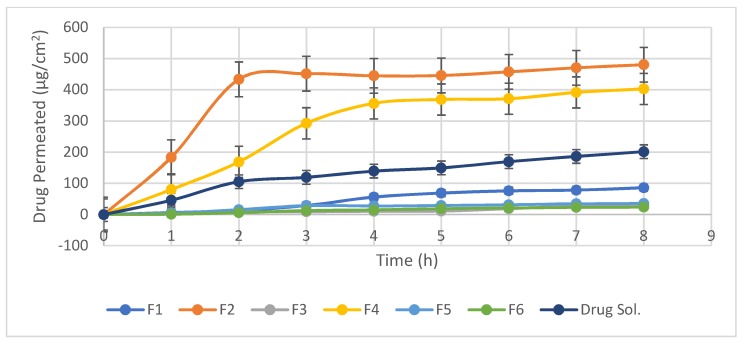
Ex-vivo corneal permeation profile of different formulae.

In general, DDAB leciplex formulae showed better permeation parameters than CTAB leciplex formulae as shown in Figure 2. These results agree with Shah et al. that found that lipid vesicles containing DDAB showed higher penetration than CTAB containing vesicles [21]. These can be explained that CTAB electrostatically interact with negatively charged phosphate group present in SPC. Therefore, lower portion of free CTAB would be available for electrostatic attraction with negatively charged corneal surface. While DDAB interact with SPC in a different manner. Hence, more DDAB could be available for the electrostatic interaction with negatively charged mucus than CTAB. These results agree with Peetla and Labhasetwar whom found that CTAB-containing polymeric nanoparticles electrostatically interact with the endothelial cell membranes, whereas in case of DDAB-containing nanoparticles, one of the alkyl chains gets incorporated in the endothelial cell model membrane [22]. It is well known that cell membranes are composed of lecithin, therefore in our study we assume that cationic surfactant (CTAB/DDAB) interact with SPC in a manner similar to its interaction with cell membrane, according to this assumption more DDAB would be available for electrostatic attraction with negatively charged sialic acid residues of corneal mucins than CTAB.

Leciplex formulae prepared without cationic surfactant (F5 and F6) showed low permeability coefficient 0.0078 and 0.00415 µg/cm, respectively compared to drug solution which showed P_app_ equal to 0.03515 µg/cm, this can be attributed to increased particle size. 

### 3.3. In-Vivo Evaluation of Carvedilol Leciplex

#### 3.3.1. Pharmacokinetic Study

**Figure 3:**
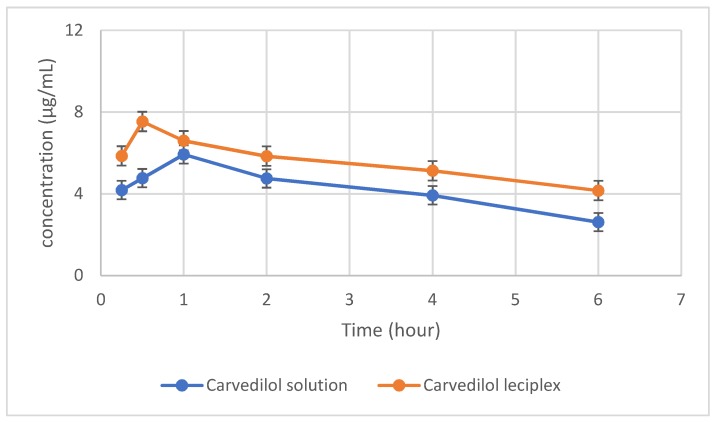
Concentration—time profiles of carvedilol in rabbit aqueous humor after single administration of different formulation.

The drug concentration–time profiles and pharmacokinetic parameters were detected after single instillation as shown in Table 4 and Figure 3. On comparing the results obtained from the two treated groups, the first group (GP1) which was treated with drug solution showed the following results: the half-life (t_1/2_) of carvedilol solution was 4.48 ± 0.8 h and the area under the curve (AUC) from 0 to 6 h was 41.79 ± 2.8 μg h/mL. Carvedilol solution had peak concentration (C_max_) of 5.93 ± 0.42 μg/mL. On the other hand, the results obtained from second group (GP2) which was treated with carvedilol leciplex showed the following parameters, AUC of 74.47 ± 1.3 μg h/mL, indicating a higher drug bioavailability. The leciplex had a peak time (t_max_) of 30 min, which was shorter than that with carvedilol solution indicating rapid onset of action. Its C_max_ was 7.53 μg/mL, which was higher than that for carvedilol solution. Carvedilol incorporated into leciplex had a t_1/2_ of 7.0 ± 0.67 h, which was 1.56 times longer than that of the carvedilol solution. Its average retention time (MRT) was 10.30 h, which was the 1.5 longer than drug solution. These results were consistent with the higher transcorneal permeability of carvedilol leciplex compared to carvedilol solution. It can be concluded from the pharmacokinetic study that the leciplex was capable of prolonging the retention time of carvedilol and enhance its bioavailability. These results are in agreement with several studies showed the effect of encapsulation the drug into nanoparticles on drug pharmacokinetics in aqueous humor where Ban et al. found that dexamethasone charged lipid nanoparticle revealed higher drug retention time and enhanced drug permeation to the cornea and consequently higher ocular bioavailability in comparison with dexamethasone aqueous solution [23]. Also, Huang et al. found that cubosomes were capable of prolonging the retention time of timolol maleate in anterior segment and aqueous humor and releasing the drug in a sustained pattern in comparison with commercial timolol eye drops [24]. 

#### 3.3.2. Pharmacodynamic Study

**Figure 4:**
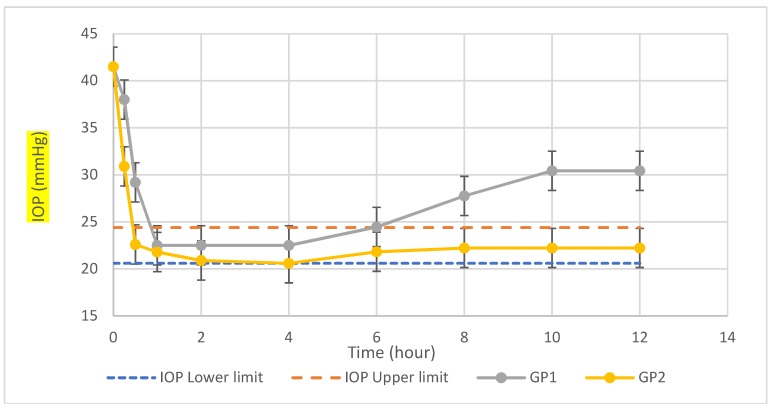
Mean IOP of hypertensive rabbits for first 12 h with different administration in comparison with physiological minimum IOP for rabbit (IOP lower limit) and physiological maximum (IOP upper limit).

Intraocular pressure was measured for each albino new zealand rabbit for 2 consecutive days and the normal IOP range was determined 20.6~24.4 mmHg. After 1-week injection of dexamethasone in the anterior chamber, IOP was increased to above 40 mmHg. The IOP of the right eye of each rabbit in both groups maintained above 40 mmHg during measurement period. For the carvedilol solution treated group (GP1), the IOP decreased to normal value (22.5 ± 2.0 mmHg) after 1 h of administration, IOP was kept in the normal range for the first six hours then IOP gradually increased as shown in Table 5 and Figure 4. For carvedilol leciplex treated group (GP2), the IOP decreased to normal value (22.6 ± 2.10 mmHg) after 30 min of administration, IOP was kept in the normal range for the 24 h then IOP gradually increased. These results were consistent with pharmacokinetic results where carvedilol leciplex formula has shorter t_max_ and longer MRT compared to carvedilol solution. These results are in agreement with Rathod and Deshpande who found that positively charged liposome containing pilocarpine nitrate showed greater duration of action and higher IOP reduction compared to negatively charged liposomes [25]. Also, Hathout et al. used 1% acetazolamide in different type of liposomal formulation and studied the IOP lowering effect in normotensive rabbits. Results showed that positively charged multilamellar vesicles of liposomes containing acetazolamide provide maximum IOP reduction compared to free drug solution and negatively charged and neutral liposomes [26]. Leonardi et al. studied encapsulation of melatonin into DDAB cationic solid lipid nanoparticle and found that cationic nanocarrier was the most effective in terms of IOP reduction compared with free drug, and its effect lasted approximately after 24 h from the instillation, whereas free drug elicited its hypotensive activity between 1 and 4 h from the application as eye drops [27].

#### 3.3.3. Histological Examination

**Figure 5:**
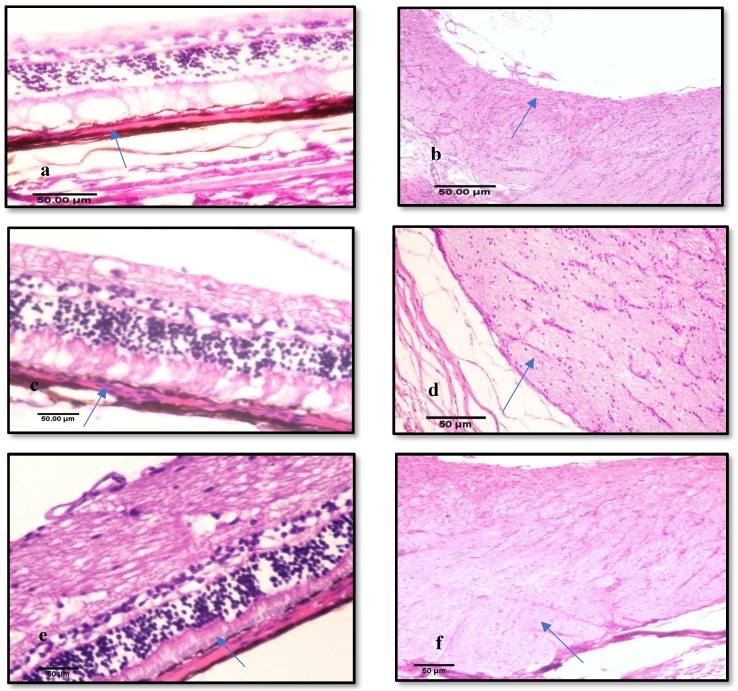
Histopathology microscopy of (**a**) Retinal atrophy with loss of retinal ganglion cells of sham group. (**b**) Optic nerve of sham group showing Wallerian degeneration and marked excavation. (**c**) Mild retinal atrophy with some loss of ganglion cells and small vacuoles after treatment with carvedilol solution (GP1). (**d**) Optic nerve showing Wallerian degeneration and without excavation after treatment with carvedilol solution (GP1). (**e**) Improvement of retinal atrophy with some loss ganglion cells and no vacuolation of choroid after treatment with carvedilol leciplex (GP2). (**f**) Optic nerve showing mild edema and thickening without excavation after treatment with carvedilol leciplex (GP2).

Histological examination of right eye of each rabbit (sham group) showed that all glaucomatous eyes of induced animals showed similar findings lesions. The hallmark of glaucoma, retinal atrophy with loss of retinal ganglion cells (RGC) and their axons. Atrophy of some layers included internal plexiform layer and internal nuclear layer were seen. Retinal atrophy, which was prominent in the inner nuclear layer and ganglion cells. Microcystoid degeneration of retina of glaucomatous eyes which characterized histologically by multiple clear round spaces found usually in the inner plexiform layer Figure 5a. Small vacuoles were usually seen in choroid which accompanied with slight thickening of retina. Optic nerve of glaucomatous eyes showed Wallerian degeneration and marked excavation or cupping and there was loss of myelin from much of the optic nerve fibers. Mild atrophy in the optic nerve were also seen Figure 5b.

Histological examination of group (GP1) which was treated by drug solution revealed that retina showed moderate retinal atrophy with some loss of internal plexiform layer and ganglion cells. Small vacuoles were seen in choroid which accompanied with slight thickening of retina Figure 5c. Optic nerve of glaucomatous eyes treated by drug solution showed Wallerian degeneration and without excavation or cupping and there was loss of myelin from much of the optic nerve fibers Figure 5d.

Histological examination of group (GP2) which was treated by carvedilol leciplex formula showed improvement of retinal atrophy of glaucomatous eyes with some loss of ganglion cells. No vacuolation of choroid was detected in treated animals group Figure 5e. Optic nerve of glaucomatous eyes showed mild edema and thickening without excavation or cupping Figure 5f. No histological changes were observed as a result of irritation or inflammation in both groups. These results are agreement with Fangueiro et al. who found that use of cationic surfactant in formulation of epigallocatechin gallate lipid nanoparticles did not cause irritation or inflammation of the eye and concluded that these lipid nanoparticles were compatible to the ocular administration [28]. This can be attributed to the results obtained by Cui et al. who demonstrated that the toxic effects of cationic surfactants are greatly diminished after association with soy lecithin [29]. 

## 4. Conclusions

The cationic leciplex nanoparticles were able to deliver the carvedilol effectively via electrostatic attraction with anionic mucin present on the cornea. Also, the nanosized of vesicles create large contact surface with ocular surface enabling enhanced corneal absorption. Based on the results of aqueous humor pharmacokinetic parameters, the selected DDAB leciplex colloidal formulation showed a significant improvement in ocular bioavailability and retention time of carvedilol compared with the drug aqueous solution. Consequently, leciplex formulation provided rapid reduction of IOP with increased duration of action compared to drug solution. Furthermore, histological examination of carvedilol leciplex treated group showed improvement of retinal atrophy of glaucomatous eyes compared to carvedilol solution treated group. These results indicated that leciplex nanoparticles are promising vectors to deliver the drug through ocular mucosa.

## Figures and Tables

**Table 1:** Composition of leciplex formulae, *n* = 3.

Formulation Code	Composition			
	SPC	CTAB	DDAB	Carvedilol
	mg/mL	mg/mL	mg/mL	mg/mL
F1	18.6	8.7	-	5
F2	18.6	-	11.1	5
F3	31	2.9	-	5
F4	31	-	3.7	5
F5	18.6	-	-	5
F6	31	-	-	5

**Table 2:** Characteristics of leciplex formulae, *n* = 3.

Formulation Code	Characteristics			
	PS	PDI	ZP	EE
	nm		mV	%
F1	91.48 ± 1.8	0.18	34.5 ± 0.58	95.10 ± 1.0
F2	16.04 ± 1.2	0.16	53.9 ± 0.91	95.59 ± 0.83
F3	706 ± 0.98	0. 47	31.6 ± 0.46	96.00 ± 0.98
F4	523 ± 1.1	0.50	47.2 ± 0.72	96.93 ± 1.3
F5	1094 ± 2.3	0.40	2.82 ± 0.54	95.27 ± 0.69
F6	1867 ± 1.5	0.70	7.83 ± 0.63	95.90 ± 0.94

**Table 3:** Transcorneal permeation parameters of different formulae, *n* = 3.

Formulae	Jss (µg/cm^2^/h)	P_app_ (µg/cm)	Q_8_ (µg/cm^2^)
F1	13.40 ± 0.36	0.0134 ± 0.060	85.80 ± 0.56
F2	115.74 ± 0.21	0.1157 ± 0.044	480.36 ± 0.58
F3	2.47 ± 0.17	0.00247 ± 0.023	27.10 ± 1.1
F4	92.49 ± 0.54	0.0924 ± 0.091	402.83 ± 0.97
F5	7.82 ± 0.29	0.0078 ± 0.023	35.53 ± 0.87
F6	4.15 ± 0.13	0.00415 ± 0.020	23.60 ± 0.80
Drug sol.	35.15 ± 0.19	0.03515 ± 0.01	201.49 ± 0.50

**Table 4:** Aqueous humor pharmacokinetic parameters of carvedilol leciplex formula and carvedilol solution.

Pharmacokinetic Parameters	Carvedilol Solution	Carvedilol Leciplex
T_1/2_ (h)	4.48 ± 0.8	7.0 ± 0.67
t_max_ (h)	1 ± 0.19	0.5 ± 0.0
C_max_ (µg/mL)	5.93 ± 0.42	7.53 ± 0.61
AUC _(0–6)_ (µg h/ML)	41.79 ± 2.8	74.47 ± 4.3
MRT (h)	6.64 ± 0.50	10.30 ± 0.96

**Table 5:** Mean intraocular pressure (IOP) value of ocular hypertensive rabbits at different time intervals with different administrations, *n* = 6.

Time (h)	Mean IOP of GP1 (mmHg)	Mean IOP of GP2 (mmHg)
0	40.9 ± 1.2	41.4 ± 1.5
0.25	38.00 ± 3.8	30.9 ± 2.1
0.5	29.21 ± 3.1	22.6 ± 2.10
1	22.5 ± 2.0	21.6 ± 1.0
2	22.5 ± 2.0	20.9 ± 0.00
4	22.5 ± 2.0	20.6 ± 0.73
6	24.4 ± 2.0	21.83 ± 2.3
8	27.7 ± 2.2	22.23 ± 2.6
10	30.4 ± 2.7	22.23 ± 2.6
12	30.4 ± 2.7	22.23 ± 2.6
24	37.0 ± 3.0	23.91 ± 3.71
48	41.7 ± 2.3	33.23 ± 1.4
